# Invasive Pneumococcal Disease: Still Lots to Learn and a Need for Standardized Data Collection Instruments

**DOI:** 10.1155/2017/2397429

**Published:** 2017-03-23

**Authors:** T. J. Marrie, G. J. Tyrrell, Sumit R. Majumdar, Dean T. Eurich

**Affiliations:** ^1^Department of Medicine, Dalhousie University, Halifax, NS, Canada; ^2^The Division of Diagnostic and Applied Microbiology, Department of Laboratory Medicine and Pathology, University of Alberta and The Provincial Laboratory for Public Health, Edmonton, AB, Canada; ^3^Department of Medicine, Faculty of Medicine and Dentistry, University of Alberta, Edmonton, AB, Canada; ^4^School of Public Health, University of Alberta, Edmonton, AB, Canada

## Abstract

*Background.* Large studies of invasive pneumococcal disease (IPD) are frequently lacking detailed clinical information.* Methods.* A population-based 15-year study of IPD in Northern Alberta.* Results.* 2435 patients with a mean age of 54.2 years formed the study group. Males outnumbered females and Aboriginal and homeless persons were overrepresented. High rates of smoking, excessive alcohol use, and illicit drug use were seen. Almost all (87%) had a major comorbidity and 15% had functional limitations prior to admission. Bacteremia, pneumonia, and meningitis were the most common major manifestations of IPD. Almost half of the patients had alteration of mental status at the time of admission and 22% required mechanical ventilation. Myocardial infarction, pulmonary embolism, and new onset stroke occurred in 1.7, 1.3, and 1.1% of the patients, respectively; of those who had echocardiograms, 35% had impaired ventricular function. The overall in-hospital mortality was 15.6%.* Conclusions.* IPD remains a serious infection in adults. In addition to immunization, preventative measures need to consider the sociodemographic features more carefully. A standard set of data need to be collected so that comparisons can be made from study to study. Future investigations should target cardiac function and pulmonary embolism prevention in this population.

## 1. Introduction

*Streptococcus pneumoniae* from the time of its discovery by Louis Pasteur in 1881 to the present day has been an important human pathogen [1]. The capsule of the pneumococcus, composed of polysaccharides, helps it avoid host defenses and hence it is a major virulence factor [2]. Challenges in finding an effective vaccine have been the number of capsular types (of which there are currently 94) and the fact that polysaccharides are not very antigenic [3]. Fortunately most of the cases of pneumococcal disease are caused by a smaller number of capsular serotypes and conjugating the polysaccharide with a protein has led to more effective vaccines [3].

There have been numerous prior studies of invasive pneumococcal disease (IPD) ranging from those with small numbers that are rich in clinical detail to those that are very large and are focused on the intended and unintended consequences of vaccination but lack necessary clinical details [4–7]. We, however, took advantage of the fact that pneumococcal protein conjugate vaccine seven (PCV-7) was introduced in Alberta, Canada, in 2002 and PCV-13 in 2010 to conduct a study of “IPD” in Northern Alberta. We specifically undertook a comprehensive and population-based study from 2000 to 2014 to provide a detailed description of invasive pneumococcal disease in a modern era. We feel this is of utmost importance as it will serve as bench mark for other studies.

## 2. Methods

### 2.1. Definitions

Cases of IPD were defined as per the national case definition of isolation of* S. pneumoniae* from a normally sterile site such as blood, CSF, pleural fluid, biopsy tissue, joint aspiration, pericardial fluid, or peritoneal fluid [8]. IPD is a provincially notifiable disease in Alberta; therefore all invasive pneumococcal isolates are submitted to the Provincial Laboratory for Public Health (PLPH) for further characterization. This allowed us to prospectively identify all cases of IPD in Northern Alberta.

### 2.2. Clinical Data Collection

Research nurses collected sociodemographic, clinical, functional, and laboratory data using a standardized case report form (CRF). The research nurses received training on data collection prior to the start of the study. In addition to the CRF, standard operating procedures documents, definitions, drug classification, and underlying illness categorization were part of their working documents. With respect to underlying illnesses, if the attending physician recorded such an illness it was accepted as such. From September 2012 through the end of the study December 31, 2014, additional data were collected for a substudy examining cardiac events during the hospital stay. Our study received approval from the institutional research review committees of the Alberta Health Regions as well as the University of Alberta ethics review board.

### 2.3. Identification and Serotyping of* S. pneumoniae* Isolates

*Streptococcus pneumoniae* isolates were received at the Provincial Laboratory of Public Health from diagnostic laboratories in Alberta as per requirements of provincial notifiable disease regulations. The isolates were confirmed as* S. pneumoniae* based on characteristic morphology and optochin susceptibility [9]. All pneumococcal isolates that exhibited a positive Quellung reaction using commercial type specific antisera obtained from Statens Serum Institute, Copenhagen, Denmark, were assigned a serotype designation. Strains that were susceptible to optochin but which failed to serotype using the Quellung assay were assayed further using AccuProbe™* Streptococcus pneumoniae* culture identification test, Gen-Probe, San Diego, CA, to confirm the species identification.

### 2.4. Comparison with Alberta Population

For comparison purposes some characteristics of the Alberta population were obtained from the Alberta Ministry of Health interactive website [10]. The representation of various occupations constituting the work force in Alberta was from [11].

## 3. Results

Two thousand four hundred and thirty-five patients with a mean age of 54.2 years had IPD over the 15 years of the study. Across all age groups, males were more likely to have IPD than females. Other sociodemographic features of the study population are given in Table 1. Noteworthy is that 7.5% were homeless and 15% had some functional limitations in the weeks prior to presentation. The occupations of those who were working are presented in Table 2. For the most part these are similar to the Alberta population. Construction work is overrepresented and 36% of this group were welders. Teachers and health care workers were not at greater risk than the general population.

Comorbid illnesses were common, being present in 98.9% (Table 3). If all cardiovascular conditions are combined, then, fully 45% of the study population were affected prior to presentation. Individually and apart from hypertension, COPD, recent cancer, and hepatitis C were most common.

The manifestations of IPD are presented in Table 4. Bacteremia and pneumonia were most common and the pneumonia patients by definition were bacteremic. Otherwise, meningitis was the most common manifestation of IPD (4.9%) while endocarditis/pericarditis were least common (<1%).

The complications and outcomes from IPD are shown in Table 5. Almost half the patients had altered mental status at the time of admission. Most (748/1039 [72%]) patients with altered mental status had a Glasgow Coma Score value recorded: only 7.5% had a score of 15 while 40.6% had a value of 10 or less with 200 having a score of 3. One-quarter of patients were admitted to ICU and 88.5% of these patients required mechanical ventilation. Most of the acute complications occurred on hospital day 1 including admission to ICU, 557/618 (90%); peritonitis, 33/39 (84%); cardiac arrest, 51/70 (73%); liver failure, 36/62 (58%); heart failure, 51/90 (57%); myocardial infarction, 44/78 (56%); renal failure requiring dialysis, 47/99 (47%).

The substudy (*N* = 811) related to cardiovascular complications is presented in Table 6. The most common complication was atrial fibrillation, which occurred in more than one in ten patients. What was surprising was that 24% had echocardiograms done, 87% of which were transthoracic, and 8.9% had severely impaired left ventricular function with ejection fractions <30–35%.

Table 7 shows the serotypes in PCV-7, PCV-13, and PPV-23 vaccines. These accounted for 22.7%, 52.3%, and 82.9% of isolates, respectively. An additional five serotypes rounded out those that accounted for 1% or more of the isolates but were not present in the first 23 serotypes listed. Overall these 28 serotypes accounted for 90.3% of the isolates. An additional 31 serotypes were represented among the remaining 11.5% of the isolates.

## 4. Discussion

The percentage of males with IPD at 56.7% was higher than the percentage of females. Despite the introduction of pneumococcal conjugate vaccines the rate of IPD among males remains higher than that among females especially in children and in adults aged 40–64 and >74 years [12]. Most of the patients with IPD had pneumonia and in studies of pneumonia in general males tend to be predominate [13].

People of Aboriginal descent were overrepresented in this study population at 12.8% whereas they constitute about 5.2% of the population of Alberta [10]. It has been noted that among resource poor populations a gap in rates of IPD remains compared with other populations in the same geographic area, a gap that will not be solved by vaccination alone [13, 14].

Almost half the patients with IPD required an ambulance to come to hospital. This is similar to what is observed at a tertiary care hospital in Halifax where 45% of the patients with all-cause pneumonia arrive by ambulance (Petrie D, personal communication). However 90% of our patients were admitted to hospital in sharp contrast to the rate of 47% admission rate for all-cause pneumonia in the Edmonton area but not surprising since most of these patients were bacteremic [15]. The 90% admission rate for patients with IPD is similar to that seen in the USA at 93% prior to introduction of PCV-13 and 95% afterwards [16] but is higher than the 66.9% admission rate among adults of 65 years of age and over with IPD in Ontario [17].

Residence at the time of admission may have implications for programs directed towards prevention of IPD in adults. Only 80% of our patients were living at home whereas 7.5% were homeless and 7.4% were in a group home setting. The rate of homelessness among patients with IPD is much higher than the 2.5% observed for patients with all-cause community-acquired pneumonia in the Edmonton area [18]. This overrepresentation of homeless persons among those with IPD is likely due to the very high rate of IPD in this population. Pleveneshi et al. found that the rate of IPD among homeless persons in Toronto was 273/100,000 per year compared with 9/100,000 per year in the general adult population [19]. One of the reasons for this high rate is crowding in homeless shelters resulting in high transmission rates of pneumococcal disease [20]. During the course of this study an outbreak of serotype 5 accounted for about a quarter of all cases and homelessness was a risk factor for transmission of serotype 5 in this outbreak [21]. The high rates of tobacco smoking and alcohol abuse in this population also contribute to the higher rates of IPD [19]. Further study is needed to determine what contribution each of these factors, homelessness, tobacco, and alcohol usage, makes to the increased risk of IPD in this population. Vaccination and other intervention programs should have a high benefit in this population.

In terms of common lifestyle risk factors for IPD, almost half of the patients in our cohort were tobacco smokers. This contrasts with 28.3% of those with all-cause pneumonia in Edmonton requiring hospitalization and with a 28% smoking rate in the general population of Alberta [18, 22]. Smoking is a well-known risk factor for IPD with odds ratio of 4.1 when compared with those without IPD [23]. There is a dose-response relationship with the number of cigarettes smoked per day and those exposed to second-hand smoke are also affected [23]. Furthermore, 25% of our patients self-reported abuse of alcohol, and alcohol abuse is well known to increase both the risk for and the severity of IPD [24–27].

Occupation may also be a risk factor for acquisition of IPD. Previously we found that welders were at increased risk for IPD with a rate of 22.7/100,000 versus 8.7/100,000 in the general adult population [28]. These findings are extended in this study. There were 53 cases among welders, accounting for 36% of all construction workers. Exposure to welding fumes promotes lung inflammation and has been shown in animal models to predispose to pneumonia [28]. Several populations with increased exposure to pneumococcal disease such as health care workers and teachers were not overrepresented. Our classification of occupation was done without reference to the National Occupational Classification Statistics Canada 2011 so some of the occupations are likely misclassified. This applies mostly to business and construction categories.

The vast majority (98.9%) of our patients had one or more comorbid illnesses. Moore et al. found that 76% of the adults with IPD in the USA had an indication for pneumococcal vaccination [16]. While comorbidities that are risk factors for IPD are well recognized and are for the most part indications for vaccination what is not readily recognized is the concept of risk factor stacking [29]. For example, when smoking is added to diabetes, chronic obstructive lung disease, and chronic heart disease the odds ratio for acquisition of IPD increases from 8.5 to >40 [29]. The highest rates of IPD are seen among those who are immunocompromised either by underlying disease or by treatment. In a Toronto study of IPD, 27.8% of the patients were immunocompromised and those with multiple myeloma had a rate of IPD of 847/100,000, for those with acute leukemia it was 220/100,000, and immunosuppressive therapy led to a rate of 20/100,000 [30]. From 2000 to 2004 we used data on IPD Alberta-wide and cancer registry data and found that compared with a rate of 11/100,000 in the general population patients with multiple myeloma had a rate of IPD of 673/100,000 and rates for those with chronic lymphocytic leukemia, acute myelogenous/acute lymphoblastic leukemia, Hodgkin's disease, and non-Hodgkin's lymphoma were 124; 129; 47; 63/100,000, respectively [31]. We also found a high rate of IPD disease in patients with lung cancer at 143.6/100,000 [31]. A finding unique to the current study is the overrepresentation of patients with hepatitis C. The implication is that hepatitis C should be added to the list of indications for pneumococcal vaccination [32].

From 1935 to present, pneumonia has remained the major manifestation of IPD [33–35]. Meningitis has been the second most common manifestation [34, 35] and endocarditis has remained uncommon at about 1% [33, 35]. Although the clinical presentation of IPD has not changed much over time, the mortality rate from IPD has declined from 77.5% in the preantibiotic era to 24.7% in the early antibiotic era to 16.9% at the turn of the 21st century and in-hospital mortality remains too high at 15.6% in our study [33–35]. Austrian and Gold noted in patients with IPD that deaths within the first 5 days seemed not to be influenced by antibiotic therapy [34] and Yu et al. in the modern era found that 64.5% of all deaths occurred in the first 3 days [35]. This is in keeping with our observation that most of the life threatening complications occurred on day 1, an indication that these patients were seriously ill on admission.

Almost half (43.7%) of the study population had alteration of mental status. This is much higher than the 11.5% rate among adults with all-cause pneumonia admitted to Edmonton hospitals [18]. It is also higher than the 23% value quoted in a review of sepsis associated encephalopathy [36]. The high rate of alteration of mental status in this study is likely accurate because 72% of the patients with this finding had a Glasgow Coma Score determined and it was abnormal in 92.5% with 40.6% having a score of 10 or less. Sepsis associated encephalopathy involves direct cellular damage to the brain [36] and is associated with abnormal electroencephalographic findings [37]. Mortality rates correlate with Glasgow Coma Score with a 63% mortality for those with a score of 3–8 [38]. This is an area which clearly needs further study.

Just over 1% of our patients had new onset stroke following admission to hospital. From 2.2 to 17% of all strokes have onset during hospitalization for a diagnosis or procedure other than stroke [39]. In a systematic review of trigger factors for ischemic stroke, Guiraud et al. found that infection within the previous week was such a trigger factor with odds ratio of 2.91 (1.41–6) and respiratory tract infection had odds ratio of 2.4 (1.2–4.8) [40]. Hospitalization for infection within 14 days was associated with an increased risk of stroke, OR 8 (1.6–77.3). In a cohort of 5639 patients followed for a median of 12.2 years, 889 developed a stroke within 14 days of hospitalization for some other illness. Twenty-nine of these patients had at least one hospitalization for infection during the preceding 90 days [41]. The infections were mainly of the respiratory or urinary tracts [41].

Pulmonary embolism occurred in 1.3% of our patients. Investigators from Taiwan found that patients with pneumococcal pneumonia were 1.97 times more likely to have a pulmonary embolus than age and sex matched controls without pneumonia [42]. There are likely several factors that predispose to pulmonary embolism in this population, namely, bed-ridden state for 3 or more days and procoagulant effect of infection [43, 44]. In a retrospective review of 1180 medical inpatients, 0.3% of those at low risk for venous thromboembolism (VTE) developed a pulmonary embolus versus 7.5% for those at high risk [45]. Thus the overall population of adult patients with IPD is at intermediate risk and should be evaluated for known risk factors for VTE and if present receive prophylaxis [46]. Clearly this is another area in need of further study.

Several of the complications that occurred were pneumonia specific. Thus decortication was necessary because of late stage empyema. Bronchopleural fistula is an uncommon complication that occurs because cavitating pneumonia results in a pneumothorax [47]. Necrotizing pneumococcal pneumonia resulting in cavitation is more common than previously appreciated. With the widespread use of computed tomographic scanning in patients with pneumococcal pneumonia 15/136 (11%) were found to have such changes [48].

While necrotizing fasciitis is more commonly associated with Group A* Streptococcus* it does occur with* S. pneumoniae* [49]. Sixty-five (2.7%) of our patients had cellulitis of whom 2 had necrotizing fasciitis.* Streptococcus pneumoniae* is secondary only to the meningococcus as a cause of purpura fulminans [50]; however to 1997 only 43 such cases had been described [51]. In contrast to meningococcemia patients with pneumococcal induced purpura fulminans are often not hypotensive and 51% to 63% are asplenic [51]. The mortality rate was 60% [49]. Our experience of 1 such case among 2435 adult patients suggests that it is uncommon but may be more common than reported in the literature.

Because of a growing literature on the cardiovascular sequelae of sepsis in general [52–57] and pneumococcal infection in particular [52–56, 58], we undertook a substudy of cardiovascular complications and risk factors part way through the study (from 2012 to 2014). Acute myocardial infarction is not uncommon following pneumonia with rates ranging from 1.5% to 15% [52, 53, 58]. However the 15% rate was in patients with severe pneumonia [53]. Rhythm disorders are also common, with 12% having a new diagnosis of cardiac arrhythmia within 90 days of admission for pneumonia [54]. Atrial fibrillation is most common, but other dysrhythmias do occur as in this study where 1.5% of patients had one or more episodes of asystole and 0.5% had ventricular tachycardia. A striking finding from our study was that 35% of patients who had an echocardiogram performed had an ejection fraction of <50% indicating systolic dysfunction. Myocardial dysfunction in sepsis has been recognized for some time and this dysfunction may be systolic or diastolic [59–61]. While several factors may play a role in the pathogenesis of cardiovascular complications during the course of IPD the pneumococcus does play a direct role. In both animal models and humans, translocation of* S. pneumoniae* into the myocardium has been observed during pneumococcal sepsis [55]. It has also been shown that pneumolysin causes microscopic lesions in the myocardium [55]. Some interventions may be beneficial; thus aspirin resulted in lower 30-day mortality following pneumonia in one study [56] and vaccination with pneumococcal polysaccharide vaccine was associated with fewer acute coronary syndrome events in another [57].

Table 7 shows selected serotyping data. Fifty-two percent of the isolates had serotypes that are in the current PCV-13 formulation. While 59 serotypes were present overall, 28 accounted for 90.3% of all cases of IPD over the 14 years of the study. The serotype shifts over the course of the study are not shown but it is apparent that more effective control of IPD in adults will require new vaccine strategies. Indeed a 15-valent PCV which contains serotypes 22F and 33F in addition to the PCV-13 serotypes is currently undergoing clinical trials [62]. Serotype 22F accounted for 7.2% of the isolates in our study about the same as the 9.8% reported Canada-wide from 2011 to 2014 [63]. Serotype 33F accounted for 3% of isolates in Canada and 1.8% in our study. The Canada-wide study included children and adults [62].

A strength of this study is the detailed collection of data which allow for a full appreciation of the various epidemiological and clinical factors in IPD. This strength is also a weakness in that in some areas necessary data were not collected. However what the study does do is allow for the development of a standardized data collection instrument which can be used in all future such studies so that comparisons from study to study can be made. There are also several noteworthy limitations to our study. First and foremost, we do not have detailed immunization data for these patients. Second, comorbidities and complications were based on physician records and not necessarily standardized definitions. For example, serial troponins were not collected on all IPD patients nor were echocardiograms done on all patients. Third, biomarkers and measures of inflammatory response were not routinely collected. Fourth, we did not gather data on the few IPD patients that may not have been admitted to hospital. Last, our cohort, though population based, was drawn from the northern half of one province in Canada and some may be concerned that our results are not generalizable to other jurisdictions or nations.

In conclusion, IPD remains a serious infection in adults. Preventative measures to improve vaccination rates especially in the elderly and groups at higher risk that may be harder to reach as shown in this study are needed [63]. Addressing sociodemographic factors that put individuals at higher risk for IPD is also necessary. A standard set of data should be collected so that comparisons can be made from study to study. Future investigations should focus on further elucidation of the cardiovascular effects of IPD and what can be done to ameliorate these effects.

## Figures and Tables

**Table 1:** Sociodemographic and lifestyle features in 2435 adults with invasive pneumococcal disease.

	Number	Percent
Number studied	2435	100
Number of males	1380	56.7
Mean age (SD)	54.2 (17.8)	
Aboriginal (first nations)	312	12.8
To hospital by ambulance	1098	45.1
Admission status		
Never seen at a hospital	3	0.1
Outpatient only with visits for IV antibiotics	10	0.4
Emergency Room only	229	9.4
Inpatient	2193	90.1
Residence prior to admission		
Home	1960	80.5
Homeless, no shelter	98	4.0
Homeless, shelter	86	3.5
Lodge/group home	119	4.9
Continuing care facility	60	2.5
Subacute care	7	0.3
Functional status in the week prior to admission		
Fully functional	2078	85.4
Walking with assistance	302	12.4
Wheelchair	37	1.5
Bedridden	18	0.7
Smoker, current	1104	45.3
Alcohol, excess use	620	25.5
Illicit drug use	482	19.8

**Table 2:** Occupations of 581 patients with IPD compared with Alberta population 2015 working in these occupations.

Occupation	IPD		Alberta population, %
	Number	%	
Accommodation, food services	37	6.3	6.5
Agriculture	22	3.7	2.2
Business	17	2.9	3.4
Construction	147	25.3	11.0
Education	35	6.0	6.7
Finance, real estate	79	13.5	4.4
Forestry, mining, oil, and gas	44	7.5	6.4
Health care	20	3.4	11.7
Professional, science, teacher	15	2.4	7.8
Transportation	30	5.1	5.9
Utilities	3	0.5	0.7

**Table 3:** Comorbid illnesses in 2435 patients with invasive pneumococcal disease.

	Number	Percent
Any underlying disease	2409	98.9
Selected underlying diseases		
Epilepsy	136	5.6
Alzheimer's disease	83	3.4
Stroke	106	4.4
Hypertension	622	25.5
Heart failure	156	6.4
Previous myocardial infarction	168	6.9
Atrial fibrillation	128	5.3
Anemia	167	6.9
Insulin dependent diabetes mellitus	87	3.6
Hepatitis C	307	12.6
Cirrhosis	151	6.2
HIV/AIDS	117	4.8
Asplenia	37	1.5
Rheumatoid arthritis	56	2.3
Asthma	266	10.9
Chronic obstructive pulmonary disease	441	18.6
Cancer within thepast 5 years	307	12.6
Lung cancer	53	2.1
Multiple myeloma	42	1.7
Chronic lymphocytic leukemia	19	0.7
Lymphoma	17	0.7
Acute leukemia	5	0.2
Solid organ transplant	14	0.6
No underlying disease	26	1.1
One underlying disease	2121	87.1
Two underlying diseases	256	10.5
Three underlying diseases	32	1.3

**Table 4:** Manifestations of invasive pneumococcal disease among 2435 patients.

Manifestation	Number	Percent
Bacteremia	2325	95.5
Bacteremia source unknown	316	12.9
Pneumonia	2009	82.2
Meningitis	120	4.9
Cellulitis	65	2.7
Sinusitis	44	1.8
Septic arthritis	40	1.6
Peritonitis	39	1.6
Otitis media	38	1.6
Endocarditis	26	1.0
Pericarditis	20	0.8

**Table 5:** Complications/outcomes of invasive pneumococcal disease among 2435 patients.

Complication	Number	Percent
Altered mental status	1039	42.7
Intensive care admission	618	25.4
Respiratory failure requiring		
Mechanical ventilation	547	22.5
Pleural effusion	846	34.7
Chest tube	255	10.5
Aspiration	179	7.4
Empyema	175	7.2
Renal failure requiring dialysis	99	4.1
Congestive heart failure	90	3.7
Myocardial infarction	78	3.2
Cardiac arrest	70	2.8
New onset seizures	67	2.8
Upper GI bleed	64	2.6
Liver failure	62	2.5
Lower GI bleed	31	1.2
New onset stroke	28	1.1
Pulmonary embolus	11	1.3
*Died (in hospital)*	*379*	*15.6*
Transferred to another acute care facility after admission	275	11.6
Remained in acute care facility after infection cured	149	6.1
Discharged home on antibiotics	1370	56.3
Home care following discharge	288	11.8

**Table 6:** Cardiac events and investigations 871 patients with IPD.

Event or investigation	Number	Percent
Atrial fibrillation	109	12.5
Myocardial infarction	15	1.7
Asystole	13	1.5
Ventricular tachycardia > 30 sec	5	0.5

Cardiology consultation	60	6.8
Coronary angiogram	9	1
Echocardiogram	210	24.1
Tricuspid regurgitation	114	54.3
Mitral regurgitation	112	53.3
Aortic regurgitation	25	11.9
Valve vegetation	5	2.3
Ejection fraction		
≥50%	170	80.7
40–45%	23	10.9
30–35%	6	2.8
20–25%	5	2.3
<20%	8	3.8

**Table 7:** Selected *Streptococcus pneumoniae* serotypes from 2401 patients (data missing for 34 patients due to nonviable isolates).

Serotype	Number	Percent
1	28	1.1
3	161	6.6
4^*∗*^	219	9.0
5	242	9.9
6A	51	2.1
6B^*∗*^	42	1.7
7F	129	5.3
9V^*∗*^	64	2.6
14^*∗*^	92	3.8
18C^*∗*^	39	1.6
19A	113	4.6
19F^*∗*^	53	2.2
23F^*∗*^	45	1.8
2	1	0.04
8	193	7.9
9N	65	2.7
10A	17	0.7
11A	80	3.3
12F	70	2.9
15B	15	0.6
17F	27	1.1
20	93	3.8
22F	176	7.2
32F	0	0
33F	45	1.8
16F	33	1.4
23A	39	1.6
23B	30	1.2
16F	33	1.4

The first 13 serotypes are in PCV-13 and first 23 in PPV-23. *∗*  denotes serotypes in PCV-7. The final four serotypes represent the remaining serotypes that accounted for ≥1% of the isolates beyond those in the 23-valent vaccine.
